# Laparoscopic Sleeve Gastrectomy: Symptoms of Gastroesophageal Reflux can be Reduced by Changes in Surgical Technique

**DOI:** 10.1007/s11695-012-0746-5

**Published:** 2012-08-23

**Authors:** Jorge Daes, Manuel E. Jimenez, Nadin Said, Juan C. Daza, Rodolfo Dennis

**Affiliations:** 1 Minimally Invasive Surgery Department, Clinica Bautista, Carrera 38 calle 71 esquina, Barranquilla, Colombia; 2 Research Department, Fundacion Cardioinfantil, Bogotá, Colombia; 3 Clinical Epidemiology Department, School of Medicine, Universidad Javeriana, Bogotá, Colombia

**Keywords:** Sleeve, Gastroesophageal reflux, Laparoscopic, Technique, Hiatal hernia

## Abstract

**Background:**

Bariatric surgery is the most effective treatment for gastro-esophageal reflux disease (GERD) in obese patients, with the Roux-en-Y gastric bypass being the technique preferred by many surgeons. Published data reporting the results of laparoscopic sleeve gastrectomy (LSG) in patients with GERD are contradictory. In a previous observational study, we found that relative narrowing of the distal sleeve, hiatal hernia (HH), and dilation of the fundus predispose to GERD after LSG. In this study, we evaluated the effects of standardization of our LSG technique on the incidence of postoperative symptoms of GERD.

**Methods:**

This was a concurrent cohort study. Patients who underwent bariatric surgery at our center were followed prospectively. LSG was performed in all patients in this series.

**Results:**

A total of 234 patients underwent surgery. There were no cases of death, fistula, or conversion to open surgery. All 134 patients who completed 6–12 months of postoperative follow-up were evaluated. Excess weight loss at 1 year was 73.5 %. In the study group, 66 patients (49.2 %) were diagnosed with GERD preoperatively, and HH was detected in 34 patients (25.3 %) intraoperatively. HH was treated by reduction in three patients, anterior repair in 28, and posterior repair in three. Only two patients (1.5 %) had symptoms of GERD at 6–12 months postoperatively.

**Conclusions:**

Our results confirm that careful attention to surgical technique can result in significantly reduced occurrence of symptoms of GERD up to 12 months postoperatively, compared with previous reports of LSG in the literature.

## Introduction

There is a strong association between morbid obesity and gastroesophageal reflux disease (GERD), with GERD occurring in approximately 50 % of morbidly obese patients. The main cause of GERD in obese patients is transient relaxation of the lower esophageal sphincter combined with increased intra-abdominal pressure [1]. Symptomatic hiatal hernia (HH) occurs in 15 % of patients with a body mass index (BMI) of >35 kg/m^2^ [2]. Bariatric surgery is the most effective treatment for GERD in obese patients, with the Roux-en-Y gastric bypass being the preferred technique by most surgeons [3–5].

Published data regarding the results of sleeve gastrectomy in patients with GERD are contradictory, and comparison between studies is difficult. Many authors do not perform laparoscopic sleeve gastrectomy (LSG) in patients with symptomatic GERD [6]. The criteria for diagnosis of GERD are not always clear, and the use of preoperative endoscopy varies among studies. Indications for LSG and the techniques used vary widely among surgeons. Symptomatic GERD has been reported to occur in 7.8–20 % of patients at 12–24 months after LSG in selected series of more than 100 patients [2, 7–9]. At the second and the third international consensus summits for sleeve gastrectomy, reflux disease was reported to occur in 6.5 % and 17 % of patients, respectively, after sleeve gastrectomy [10, 11].

A recent review of the literature found that four studies reported an increase in reflux symptoms after sleeve gastrectomy, and seven studies reported a reduction in symptoms [12]. Most studies reported an increase in reflux symptoms during the first year following sleeve gastrectomy, followed by a gradual decrease in symptoms up to the third postoperative year [13].

We have performed more than 1,300 sleeve gastrectomies since 2006. In a previous observational study, we identified three technical problems which explained most cases of GERD and some cases of weight regain after LSG. These problems were: a relative narrowing at the junction of the vertical and the horizontal portions of the sleeve, a dilated fundus, and a persistent HH. Some of these problems have previously been discussed in the literature [14]. When we started removing more of the fundus of the stomach (leaving only enough for oversewing of the staple line), routinely correcting the HH, and avoiding narrowing or torsion of the sleeve, we observed a substantial decrease in the need for postoperative endoscopy to investigate food intolerance or symptoms of GERD.

This study prospectively evaluated the effects of standardization of the LSG technique as described above on the incidence of postoperative symptoms of GERD. The other objectives were to establish the preoperative prevalence of GERD, esophagitis, and HH; the morbidity associated with the LSG procedure; the percentage of excess weight loss at 1 year; and the correlation between preoperative endoscopic diagnosis of HH and intraoperative findings.

## Materials and Methods

We prospectively followed all patients who underwent bariatric surgery from April 2011 to April 2012. All these patients underwent LSG. Patients with HH and reflux disease were included in the study, including two patients who had a band removed and one patient with ultra-short-segment Barrett's disease. All patients completed a multidisciplinary preoperative evaluation and met the criteria for LSG of the Colombian Surgical Society.

Preoperative GERD was diagnosed if patients experienced symptoms of heartburn or acid reflux more than twice a week, if they required antacid treatment for more than 2 weeks, or if preoperative endoscopy showed esophagitis.

All patients underwent preoperative endoscopy by an endoscopist/surgeon with more than 25 years of experience. A small HH was diagnosed when there was a patulous cardia or a hernia measuring less than 2 cm. A large HH was diagnosed when there was a hernia measuring more than 2 cm. The presence and degree of esophagitis and Barrett's disease were recorded together with any additional pathological findings. All operations were performed by our group.

### Surgical Procedure

We use five ports when performing LSG. A 12-mm port is placed at the umbilicus for insertion of the laparoscope, stapling of the stomach, and eventual removal of the stomach. A second 12-mm port is placed at the left flank for devascularization of the greater curvature of the stomach, as a secondary position for insertion of the laparoscope during stapling of the stomach, and for suturing. Three additional 5 mm ports are placed: one at the epigastrium for liver retraction, one at the left upper quadrant for the surgeon's left hand working trocar, and one at the left lateral subcostal area for the assistant.

After emptying the stomach, the greater curvature is devascularized using an ultrasonic device, starting 3 cm proximal to the pylorus and continuing until the fundus is dissected free from the left crus of the diaphragm. We ensure that there is at least 3 cm of intrabdominal esophagus, which sometimes requires dissection of the peri-esophageal fat pad. When a HH is present, we completely free the esophageal-gastric union from the left and right crura, divide the phrenoesophageal membrane and peri-esophageal connective tissues, and continue the dissection well into the mediastinum to ensure a sufficient length of intra-abdominal esophagus. The HH defect is then closed with non-absorbable monofilament sutures, anterior or posterior to the esophagus depending on the size of the defect and the resulting position of the esophagus. We do not use a calibrating bougie for hiatal closure and do not use mesh even for large hernias.

A 32 Fr bougie is introduced to reach the distal antrum. Division of the stomach starts 3 cm proximal to the pylorus, making sure that the bougie stays adjacent to the lesser curvature of the stomach. We use a 60–4.8 mm cartridge for the initial firing of the stapler, followed by three to five 60–3.5 mm cartridges. We take extra care to avoid relative narrowing at the junction between the vertical and horizontal parts of the stomach, which usually occurs during the first and occasionally the second firing of the stapler if it is inappropriately pressed against the bougie in an attempt to leave a small antrum. Narrowing can be avoided by using an articulating stapler, slightly angled to the greater curvature, to create a wide angle at the junction between the horizontal antrum and the vertical body of the stomach.

In some cases with the pylorus located in the upper right quadrant of the abdomen and a very curved stomach, the only way to form a small antrum and avoid relative narrowing is to place an additional trocar through a port at the right flank to help aim the first stapler in the correct direction. We also emphasize that when dividing the stomach, the same distance should be maintained between the lesser curvature and the entire staple line to avoid twisting of the sleeve, which may result in food regurgitation, vomiting, or GERD. Division of the stomach, including most of the fundus, is then completed, leaving only a small portion for oversewing.

We routinely bury the stapler line with continuous sero-serosal stitches using non-absorbable monofilament sutures, starting at the top of the sleeve. We then retract the hollow bougie proximally and test the sleeve with methylene blue to confirm complete and uniform filling. Adequate distention occurs with 25–35 ml. We then perform a small omental patch with a single sero-serosal stitch over the top of the sleeve. We carefully extract the removed stomach through the umbilical port after incising the aponeurosis without the use of bags.

We routinely instill diluted bupivicaine into the left upper quadrant of the abdomen. We do not use drains. The trocars are removed under direct vision to ensure hemostasis. The incised aponeurosis at the umbilical port is carefully sutured. The technical aspects described can be viewed at http://www.sages.org/video/details.php?id=103375.

The patient is discharged the following day after tolerating a liquid diet and receiving a complete set of instructions which includes aspects of nutrition, medication, activity, appointments, and mental health. The nurse coordinator delivers and explains the instructions to the patient.

The patient is evaluated on the eighth postoperative day and then at 1, 3, 6, and 12 months postoperatively and twice a year thereafter. We record symptoms of GERD using the same survey as in the preoperative evaluation. Early endoscopy is indicated if the patient has difficulty tolerating solid food and after the sixth postoperative month in patients with symptoms of GERD.

### Statistical Analysis

A Microsoft Access database (Office 2000) was used for data collection. All statistical analyses were performed using Epi Info 7.0 software (Centers for Disease Control and Prevention,Atlanta,GA).A two-sided *p* value of <0.05 was considered significant. Bivariate analyses used the *χ* ^2^ test or Fisher's exact test for categorical data and the independent-sample *t* test for continuous data.

## Results

A total of 234 patients underwent surgery during the 12-month study period. This report focuses on the 134 patients who have completed 6–12 months of follow-up, because reflux symptoms in the first postoperative months may represent an adaptation to the restricted stomach size. Table 1 shows the characteristics of patients in the total cohort (*n* = 234) and the cohort analyzed in this study (*n* = 134). Most patients were severely obese young females, and about half had both HH and GERD.

**Table 1:** Characteristics of patients who underwent LSG: total cohort and study cohort

Characteristic	Total cohort (*n* = 234)	Study cohort (*n* = 134)
Age in years (SD)	36.4	37.8
Female (%)	139 (59)	87 (65)
BMI, kg/m^2^ (range)	39 (30–64)	38 (30–64)
GERD (%)	112 (48)	66 (49)
Hiatal hernia (%)	117 (50)	65 (49)

There were no cases of death, fistula, or conversion to open surgery. One patient developed postoperative bleeding requiring revision surgery, but the cause of the bleeding was not identified (Table 2). Four patients complained of difficulty tolerating solid food in the early postoperative period. This difficulty resolved completely in three of the four patients after endoscopy alone, and the other patient had slight torsion of the distal sleeve which improved after endoscopic balloon dilatation.

**Table 2:** Surgical findings and outcomes: total cohort and study cohort

Outcome	Total cohort (*n* = 234)	Study cohort (*n* = 134)
Perioperative death	0	0
Fistula	0	0
Bleeding	1	1
Hiatal hernia, confirmed (%)	58 (25)	34 (25)
Hiatal hernia, large (%)	21 (9)	12 (9)

Interestingly, only a fraction of HHs diagnosed preoperatively were confirmed intraoperatively, and of the 69 patients without HH diagnosed on preoperative endoscopy, six had HH detected intraoperatively. HH was detected intraoperatively in 34 patients (25 %) in the study cohort, of which 29 (85.3 %) had preoperative symptoms of GERD. Twelve patients had a large HH, of which all had preoperative symptoms of GERD. The HH was reduced in three patients, repaired anteriorly in 28 patients, and repaired posteriorly in three patients.

Table 3 shows the preoperative characteristics of patients with and without GERD. There were significant differences in BMI and in the frequency of diagnosis of HH and large HH between these two groups (*p* < 0.01). HH was six times more frequent in patients with GERD than those without, and large HH only occurred in patients with GERD.

**Table 3:** Baseline characteristics of patients with and without GERD: study cohort (*n* = 134)

Characteristic	With GERD (*n* = 66)	Without GERD (*n* = 68)
Age in years*	37.6	37.9
Female (%)*	43 (65)	44 (65)
BMI, kg/m^2^**	39.3	36.5
Hiatal hernia (%)**	29 (44)	5 (7)
Hiatal hernia, large (%)**	12 (18)	0

*Differences not statistically significant, **p < 0.01

At the 6–12 months postoperative follow-up, only two patients (1.5 %) had symptoms of GERD. Both were evaluated endoscopically and were found to have a small HH without esophagitis. These two patients are currently treated with proton pump inhibitors. Both these patients had a large HH on preoperative endoscopy. Noteworthy is the fact that no patient with small or no HH was found to have postoperative symptoms (*p* = 0.021) (Table 4).

**Table 4:** Characteristics of patients with GERD at follow-up, compared with all other patients in the study cohort

Characteristic	GERD (patient 1)	GERD (patient 2)	No GERD (*n* = 132)
Age (years)	53	27	37.3
Sex	Female	Female	87M, 47F
BMI (kg/m^2^)	47	34	38
Hiatal hernia	Yes	Yes	32 (24 %)
Hiatal hernia, large	Yes	Yes	10 (7.5 %)
Reduction of excess weight	58.4 %	81 %	73.5 %

## Discussion

The results of this study show a very low incidence of GERD (1.5 %) at 6–12 months after LSG which was performed with careful attention to the described technical details. These results are even more significant because this was an unselected cohort of patients, with no exclusions. The prevalence of GERD in this series is similar to that reported in the literature, but the mean BMI is slightly lower. There were no cases of death, fistula, or conversion to open surgery. The morbidity was very low and the excess weight loss at 1 year was 74 %.

There is a strong association between morbid obesity and GERD, with GERD occurring in approximately 50 % of morbidly obese patients. There is a stronger association between GERD and waist circumference (a marker of central adiposity) than between GERD and BMI [15]. The main cause of GERD in obese patients is transient relaxation of the lower esophageal sphincter combined with increased intra-abdominal pressure [1].

Bariatric surgery is the most effective treatment for GERD in obese patients, with the Roux-en-Y gastric bypass being the preferred technique by most surgeons [3–5]. Our preferred bariatric surgery procedure has changed from the adjustable gastric band and the Roux-en-Y gastric bypass in the period from 1999 to 2005 to the LSG. The reasons for this change are explained in Table 5. The great majority of our patients prefer the LSG.

**Table 5:** Advantages of laparoscopic sleeve gastrectomy

1. Low morbidity and mortality rates.
2. Effective in the medium term.
3. Excellent resolution of co-morbidities including type 2 diabetes mellitus.
4. No marginal ulceration, internal hernia, or nutritional deficiencies.
5. Enables full endoscopic examination of the stomach postoperatively.
6. Easily revised or converted.
7. Short operation and hospitalization times.

Published data regarding the effects of sleeve gastrectomy in patients with GERD are contradictory, and comparison between studies is difficult. The criteria for diagnosis of GERD are not always clear, and the use of preoperative endoscopy varies among studies. Many authors routinely exclude patients with symptomatic GERD as candidates for LSG. Indications for LSG and the surgical techniques used vary widely among authors.

In selected series involving more than 100 LSG patients, symptomatic GERD was reported to occur in 7.8–20 % of patients at 12– 24 months postoperatively. Cottan et al. [7] reported a series of 126 patients who underwent LSG and found a 20 % incidence of GERD at 12 months postoperatively. Hamoui et al. [8] reported 131 LSG patients with a 12.7 % incidence of GERD at 13 months, Nocca et al. [9] reported 163 LSG patients with an 11.8 % incidence of GERD at 24 months, and Sorelly et al. [2] reported 264 LSG patients with a 7.8 % incidence of GERD at 24 months.

Some postoperative factors tend to reduce reflux after sleeve gastrectomy, such as reduced intra-abdominal pressure due to decreased body weight, reduced acid production, and accelerated gastric emptying (which has been well demonstrated in scintigraphy studies) [16, 17]. However, many postoperative factors predispose to reflux, such as lack of gastric compliance (which is the objective of the procedure), increased intraluminal pressure with an intact pylorus, low esophageal sphincter pressure (which has been described in a recent manometric study and is attributed to resection of the sling fibers of the distal part of the lower sphincter) [16], and the final shape of the sleeve. The shape of the superior pouch and the tubular configuration contribute to reflux but not the configuration of the inferior pouch [13].

We identified three technical errors that explain most of our cases of GERD after sleeve gastrectomy: relative narrowing at the junction of the vertical and horizontal parts of the sleeve, dilation of the fundus, and persistence of the HH or a patulous cardia.

We have performed over 1,300 sleeve gastrectomies since 2006. Early in our experience, 18 patients developed severe reflux symptoms, food intolerance, and vomiting or nausea postoperatively. Endoscopy revealed narrowing of the sleeve in these patients, and all responded to several endoscopic balloon dilatations and medical therapy. Some of these patients had prolonged symptoms, but none required additional surgery. We believe that the narrowing was due to a technical error resulting from excessive tension on the lesser curvature of the stomach when attempting to place the bougie closer to the lesser curvature during the first or second firing of the stapler. Another cause of partial obstruction in the distal part of the stomach was torsion of the sleeve, which can be prevented by maintaining the same distance between the lesser curvature and the entire staple line during division of the stomach.

Later in our experience, four patients developed symptoms of GERD after LSG. All these patients had HH, dilation of the fundus, and weight regain. We performed remedial operations in all these patients, including repair of the HH and repeat formation of the sleeve, with excellent symptomatic relief and no complications.

When we started to remove the fundus of the stomach more completely (leaving only enough to allow oversewing), routinely corrected the HH, and avoided relative narrowing or torsion of the sleeve, we observed a sharp decrease in the need for postoperative endoscopy to investigate food intolerance or symptoms of GERD.

The two patients in this series with persistent symptoms of GERD both had a large HH on preoperative endoscopy and a small HH on postoperative endoscopy. This raises the question of the need for using mesh in such cases, as has been suggested in the literature [18–20]. The development of improved biological meshes might facilitate their use in this situation. There was a surprising difference in the diagnosis of HH on preoperative endoscopy compared with intraoperative findings. One plausible explanation for this is that small hernias may have been reduced during dissection and pulling of the fundus to free it from the left crus of the diaphragm.

Our study has several limitations. First, postoperative endoscopy was not performed routinely, but only in patients with symptoms of reflux. The study was designed to evaluate symptomatic GERD, but routine postoperative endoscopy can detect recurrent HH. Second, we did not have a concurrent control cohort of patients who underwent a different surgical procedure, so our results can only be compared with those in the literature. However, the results of this study may be used as a basis for such an investigation. Third, our surgical team has substantial experience over time with LSG, and results may differ in other cohorts. Finally, we still need to evaluate longer term outcomes (more than 12 months postoperatively).

## Conclusions

Our study confirms a substantial prevalence of GERD symptoms and HH in obese patients who have undergone LSH. Our results show that careful attention to surgical techniques can significantly reduce the frequency of postoperative symptoms of GERD up to 12 months after surgery, compared with previous reports of LSG.
